# Cost-Effectiveness Analysis of Adding Daratumumab to Bortezomib, Melphalan, and Prednisone for Untreated Multiple Myeloma

**DOI:** 10.3389/fphar.2021.608685

**Published:** 2021-03-01

**Authors:** Yaohua Cao, Lina Zhao, Tiantian Zhang, Weiling Cao

**Affiliations:** ^1^ Department of Pharmacy, The Third Affiliated Hospital (The Affiliated Luohu Hospital) of Shenzhen University, Shenzhen, China; ^2^ College of Pharmacy, Jinan University, Guangzhou, China; ^3^ Guangzhou Huabo Biopharmaceutical Research Institute, Guangzhou, China

**Keywords:** Markov, cost-effectiveness, daratumumab, multiple myeloma, not eligible for stem cell transplantation

## Abstract

**Background:** To evaluate the cost-effectiveness of adding daratumumab to bortezomib, melphalan, and prednisone for transplant-ineligible newly diagnosed multiple myeloma patients.

**Methods:** A three-state Markov model was developed from the perspective of US payers to simulate the disease development of patient’s life time for daratumumab plus bortezomib, melphalan, and prednisone (D-VMP) and bortezomib, melphalan, and prednisone (VMP) regimens. The primary outputs were total costs, expected life-years (LYs), quality-adjusted life-years (QALYs) and incremental cost-effectiveness ratios (ICERs).

**Results:** The base case results showed that adding daratumumab to VMP provided an additional 3.00 Lys or 2.03 QALYs, at a cost of $262,526 per LY or $388,364 per QALY. Sensitivity analysis indicated that the results were most sensitive to utility of progression disease of D-VMP regimens, but no matter how these parameters changed, ICERs remained higher than $150,000 per QALY.

**Conclusion:** In the case that the upper limit of willingness to pay threshold was $150,000 per QALY from the perspective of US payers, D-VMP was not a cost-effective regimen compared to VMP.

## Introduction

Multiple myeloma (MM) is the second most common hematological malignancy in the world after non-Hodgkin’s lymphoma (Zou et al., 2013). According to the 2020 statistics of Surveillance, Epidemiology, and End Results Program, there were approximately 32,270 new diagnosed cases of MM and 12,830 MM-related deaths every year in the United States. MM accounted for 1.8 and 2.1% of all new cancer cases and all cancer deaths, respectively. The rate of new cases of myeloma was 7.0 per 100,000 men and women per year. The death rate of MM was 3.3 per 100,000 men and women per year. There were still approximately 140,779 people suffering from myeloma in 2017, and the number of patients was on the rise (SEER, 2020). Median survival of MM is only 5 years. It is more prevalent in the older population. Therapies of MM is constantly improving, from proteasome inhibitors to immunomodulatory drugs to targeted therapies. Although progress in the treatment of MM has been made, the disease remains incurable (Mikhael et al., 2019).

Bortezomib, melphalan, and prednisone (VMP) is one of the standard treatments for transplant-ineligible newly diagnosed multiple myeloma (TNE NDMM) (San Miguel et al., 2008; Palumbo et al., 2010; Benboubker et al., 2014; Cao et al., 2019). Daratumumab-based combinations reduced the risk of disease progression or death by more than 60% in CASTOR trial (Palumbo et al., 2016) and POLLUX trial (Dimopoulos et al., 2016b). Therefore, Mateos et al. established a new treatment option - combined daratumumab and VMP - daratumumab plus bortezomib, melphalan and prednisone (D-VMP) (Mateos et al., 2018). In the updated guidelines, D-VMP regimen was recommended to treat TNE NDMM (Kumar et al., 2019). Daratumumab is a human IgGκ monoclonal antibody against CD38. Daratumumab binds the CD38 molecule and mediates tumor cell killing through mechanisms including complement dependent cytotoxicity (CDC), antibody-dependent cellular phagocytosis, antibody-dependent cellular cytotoxicity (ADCC), and direct induction of tumor cell apoptosis (Wang et al., 2018). and it was current approved by US Food and Drug Administration (FDA) (Dimopoulos et al., 2016a; Dimopoulos et al., 2016b; Moreau et al., 2016; Palumbo et al., 2016). D-VMP significantly improved progression-free survival (PFS) and overall survival (OS) compared to VMP. D-VMP reduced the risk compared with VMP by 58% (hazard ratio [HR], 0.42; 95% confidence interval [CI], 0.34 to 0.51; *p* < 0.0001) and 40% (HR, 0.60; 95% CI, 0.46 to 0.80; *p* < 0.0003) of progression and death (Mateos et al., 2020).

D-VMP conferred patients with substantial improvement in health outcomes, but its cost was much higher than VMP. Therefore, we did this study to evaluate the cost-effectiveness of D-VMP and VMP to treat TNE NDMM patients from the perspective of US payers.

## Materials and Methods

### Model

**FIGURE 1:**
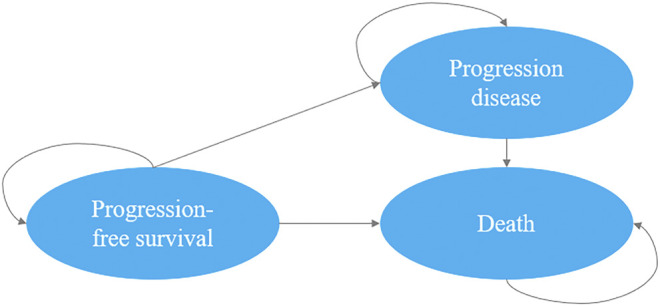
Markov state transition model.

We developed a decision-analytic Markov model which included three mutually exclusive health states: PFS state, progression disease and death. In our study, all patients (350 patients in d-VMP group and 356 patients in VMP group) were simulated from the PFS state, the patient may then progress or die. As the progression state, according to the update analysis of ALCYONE, both groups could receive subsequent line(s) of active treatment or best supportive care until death (Mateos et al., 2020). The Markov cycle length was set as one month, the initial age was set as the median age of the population and the time horizon was life time. We used a discount rate of 3% on costs and results from the perspective of US payers (Wan et al., 2019). The upper limit of willingness to pay (WTP) threshold for per quality-adjusted life-years (QALYs) was $150,000 (Neumann et al., 2014). The primary outputs of interest generated by the model included total costs, expected life-years (LYs), QALYs and incremental cost-effectiveness ratios (ICERs). The Markov model was implemented by using TreeAge Pro 2020 Software, and we used R software version 4.0.2 for statistical analysis (Figure 1).

### Cost Estimates

We considered only direct medical care costs, including drug, evaluation and management, treatment costs of adverse events (AEs). The drug unit cost was obtained from Centers for Medicare and Medicaid Services (CMS), costs of AEs were used the data from published literature (Zhang et al., 2018; CMS, 2020b; REDBOOK, 2020). And we used the US consumer price index to adjust the cost of inflation to reflect the 2020 US dollars. We assumed that the patient’s average body weight and body surface area (BSA) are 79 kg body mass and 1.91 square meter, which were met with previously published literature data (Pelligra et al., 2017). The most common treatment-related serious adverse events (SAEs) of grade 3 or 4 were included in our model, respectively is neutropenia, thrombocytopenia, anemia, pneumonia. The subsequent treatments costs of each group after progression were estimated by weighted according to the proportion of patients received regimens.

As for evaluation and management, we consulted clinicians about the specific use duration and usage of these drugs in clinical application, and calculated the management costs of the corresponding drugs and laboratory tests according to the calculation formula in the Medicare Fee-for-Service Payment and of CMS (CMS, 2020a; CMS, 2020c; Table 1).

**TABLE 1:** Key model parameters.

Parameter	Base case	95% CI or range	PSA Distribution	References
Drug cost/$				
Daratumumab 10 mg	56.063	±20%	Gamma	CMS CMS (2020b)
Bortezomib 0.1 mg	24.167	±20%	Gamma	CMS CMS (2020b)
Prednisone 1 mg	0.012	±20%	Gamma	CMS CMS (2020b)
Dexamethasone 0.25 mg	0.103	±20%	Gamma	CMS CMS (2020b)
Paracetamol 650 mg	0.037	±20%	Gamma	REDBOOK REDBOOK (2020)
Diphenhydramine 50 mg	0.017	±20%	Gamma	REDBOOK REDBOOK (2020)
Melphalan 2 mg	9.159	±20%	Gamma	REDBOOK REDBOOK (2020)
Lenalidomide 25 mg	763.010	±20%	Gamma	REDBOOK REDBOOK (2020)
Carfilzomib 1 mg	38.972	±20%	Gamma	CMS CMS (2020b)
PD cost for D-VMP	17,766.986	±20%	Gamma	Estimated Mateos et al. (2018); CMS (2020b); Mateos et al. (2020)
PD cost for VMP	16,440.419	±20%	Gamma	Estimated Mateos et al. (2018); CMS (2020b); Mateos et al. (2020)
Administration/$				
D-VMP/every infusion	349.821	±20%	Gamma	Estimated CMS (2020c)
VMP/every injection	49.928	±20%	Gamma	Estimated CMS (2020c)
Test or Monitoring/$				
Laboratory tests	36.145	±20%	Gamma	Estimated CMS (2020a)
Monitoring for PD	481.695	±20%	Gamma	Usmani SZ et al. Usmani et al. (2016)
AE related/$				
AE cost of D-VMP/per cycle	240.318	±20%	Gamma	Estimated Mateos et al. (2018); Zhang et al. (2018); Mateos et al. (2020)
AE cost of VMP/per cycle	621.084	±20%	Gamma	Estimated Mateos et al. (2018); Zhang et al. (2018); Mateos et al. (2020)
Survival & Utilities				
HR of PFS	0.42	0.34–0.51	Beta	ALCYONE Mateos et al. (2020)
HR of OS	0.60	0.46–0.80	Beta	ALCYONE Mateos et al. (2020)
PFS for D-VMP	0.685	±20%	Beta	Estimated Pelligra et al. (2017); Zhang et al. (2018); Gong et al. (2019); Hatswell et al. (2019)
PFS for VMP	0.627	±20%	Beta	Hatswell AJ et al. Hatswell et al. (2019)
PD for D-VMP	0.59	±20%	Beta	Usmani SZ et al. Usmani et al. (2016)
PD for VMP	0.59	±20%	Beta	Usmani SZ et al. Usmani et al. (2016)
Others				
Body weight	79.0	49.8–199.0	Gamma	Gong CL et al. Gong et al. (2019)
Body surface area	1.91	1.40–2.53	Gamma	Gong CL et al. Gong et al. (2019)
Discount factor	3%	0%–5%	—	Wan X et al. Wan et al. (2019)

PD, progression disease; D-VMP, daratumumab plus bortezomib, melphalan and prednisone; VMP, bortezomib, melphalan and prednisone; AE, Adverse event; HR, hazard ratio; PFS, progression-free survival; OS, overall survival; CI, confidence interval; PSA, probabilistic sensitivity analysis; CMS, Centers for Medicare and Medicaid Services.

### Survival Curve Estimation

We used the approach described by Hoyle and Henley (Hoyle and Henley, 2011) to extract data points of VMP regimens from the PFS and OS survival curves of ALCYONE (Mateos et al., 2020). First, R software (version 4.0.2) was used to extract the graphical data from the PFS and OS Kaplan–Meier curves of D-VMP and VMP, then we reconstructed individual patient data (IPD) and used the extracted values and number at risk to simulate through Weibull, log-normal, log-logistic, logistic to select the best simulation curve based on the Akaike information criterion (AIC) and Bayesian information criterion (BIC) values (Dimopoulos et al., 2007; Weber et al., 2007). The Weibull distribution was the best fitting method for our study. The transition probability of VMP regimen was obtained by Weibull survival function S(t) = exp(−λt^γ^). Then calculated the transition probability of D-VMP regimen according to HR (D-VMP vs. VMP). We estimated the probability of death in each age background based on the 2017 US life Table (Arias, 2019; Table 2).

**TABLE 2:** Parametric survival distributions.

Parametric model	PFS		OS	
	AIC	BIC	AIC	BIC
Weibull	2329.102	2336.858	1466.894	1474.638
Lognormal	2381.626	2389.382	1501.143	1508.888
Loglogistic	2355.820	2363.576	1478.216	1485.960
Logistic	2423.814	2431.570	1513.712	1521.457

PFS, progression-free survival; OS, overall survival; AIC, Akaike information criterion; BIC, Bayesian information criterion.

### Utility Estimates

The quality of life (QOL) is often referred to as utility. It is usually calculated based on the results obtained from EuroQol five dimensions questionnaire (EQ-5D) and Treatment of Cancer Quality of Life Questionnaire C30 (EORTC QLQ-C30). For PFS, Hatswell AJ et al. (Hatswell et al., 2019) used meta-regression to analyze the health state utilities of MM, found that mean first-line utility of MM was 0.627, then we used this value for VMP group. There was no utilities data for daratumumab-based regimens to treat TNE NDMM. We summarized utilities related to daratumumab-based regimens in several studies for relapsed or refractory multiple myeloma (RRMM) (Pelligra et al., 2017; Zhang et al., 2018; Gong et al., 2019), and combined the mean first-line utility of MM to estimate the utility of D-VMP group. For the utility of progression disease, we assumed the same value (Usmani et al., 2016; Table 1).

### Sensitivity Analysis

In order to evaluate the stability of the model and solve the uncertainty of the input parameters, it is generally necessary to carry out one-way sensitivity analysis and probabilistic sensitivity analysis (PSA).

For one-way sensitivity analysis, the lower limit and upper limit used between bounds of their 95% CIs to determine the impact of key parameters on the model. If there no 95% CI values, it will be changed according to the published literature or varied by ±20% of the parameters.

For PSA, we conducted 1,000 replicated Monte Carlo simulations to test the effect of changing all parameters of the model simultaneously on our outcomes. Statistical distribution sampling based on parameter characteristics. This result was used to plot the cost-effectiveness acceptability curves and scatter plot (Table 1).

## Results

### Base Case Results

The model simulated the disease development of patient’s life time for D-VMP and VMP regimens. For TNE NDMM patients, adding daratumumab to VMP provided an additional 3.00 LYs. Accounting for QOL, patients in D-VMP group gained 6.40 QALYs; this was 2.03 QALYs more than for patients in VMP group. Compared with the VMP strategy, the mean incremental costs of the D-VMP were $788,541; the incremental cost per QALY gained was $388,364; the incremental cost per LY gained was $262,526 (Table 3).

**TABLE 3:** Base case results.

Regimen	LYs	QALYs	Cost	ICER	
				per LY	per QALY
D-VMP	10.300	6.404	$1,927,635	$262,526	$388,364
VMP	7.296	4.373	$1,139,094		

D-VMP, daratumumab plus bortezomib, melphalan and prednisone; VMP, bortezomib, melphalan and prednisone; LY, life-year; QALY, quality-adjusted life-year; ICER, incremental cost-effectiveness ratio.

### Sensitivity Analysis

**FIGURE 2:**
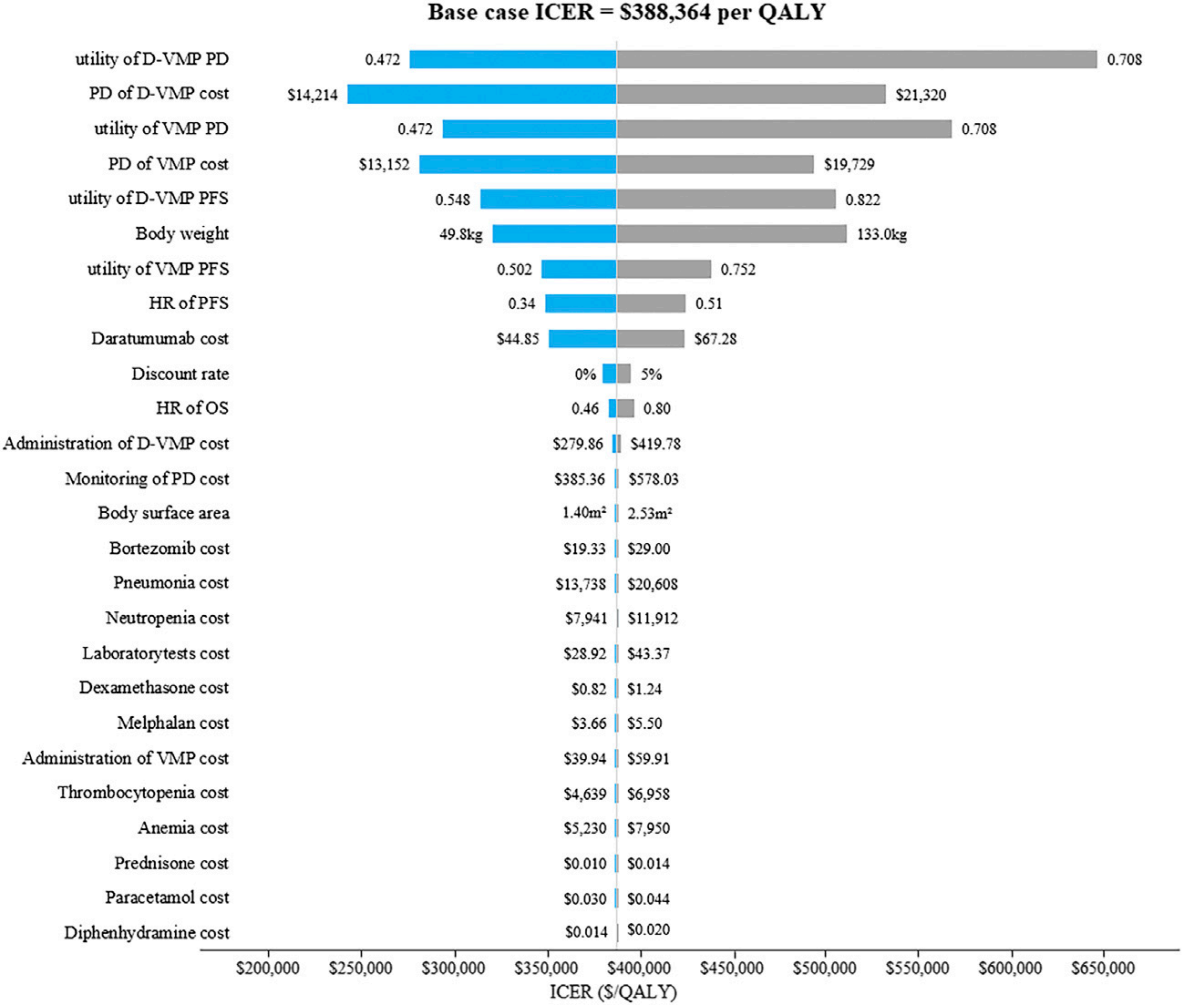
The tornado diagrams of one-way sensitivity analysis. D-VMP: daratumumab plus bortezomib, melphalan and prednisone; VMP: bortezomib, melphalan and prednisone; PD: progression disease; HR: hazard ratio; PFS: progression-free survival; OS: overall survival. ICER: incremental cost-effectiveness ratio; QALY: quality-adjusted life-year.

The results of one-way sensitivity analysis were showed in Figure 2. The descending order of factors affecting ICER were displayed from top to bottom. The tornado diagrams showed that ICER was more sensitive to the utility of progression disease in the D-VMP group, the utility of progression disease in the VMP group, the cost of progression disease in the D-VMP group, the weight of patient and the cost of progression disease in the VMP group. But no matter how these parameters changed, ICERs remained higher than WTP.

**FIGURE 3:**
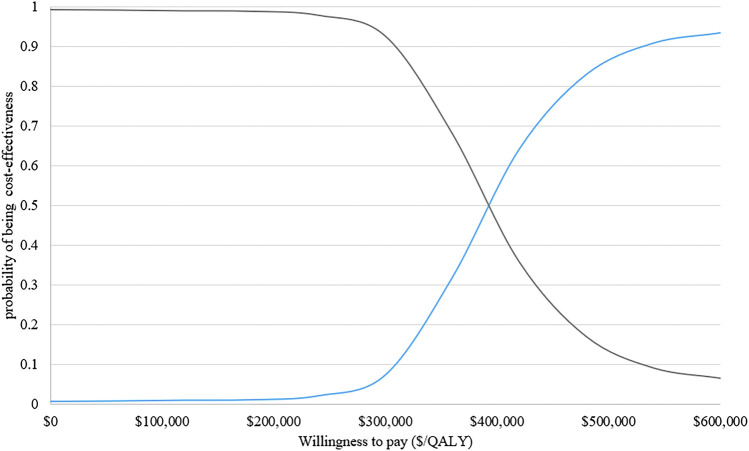
Cost-effectiveness acceptability curves of D-VMP vs. VMP. QALY: quality-adjusted life-year; D-VMP: daratumumab plus bortezomib, melphalan and prednisone; VMP: bortezomib, melphalan and prednisone.

**FIGURE 4:**
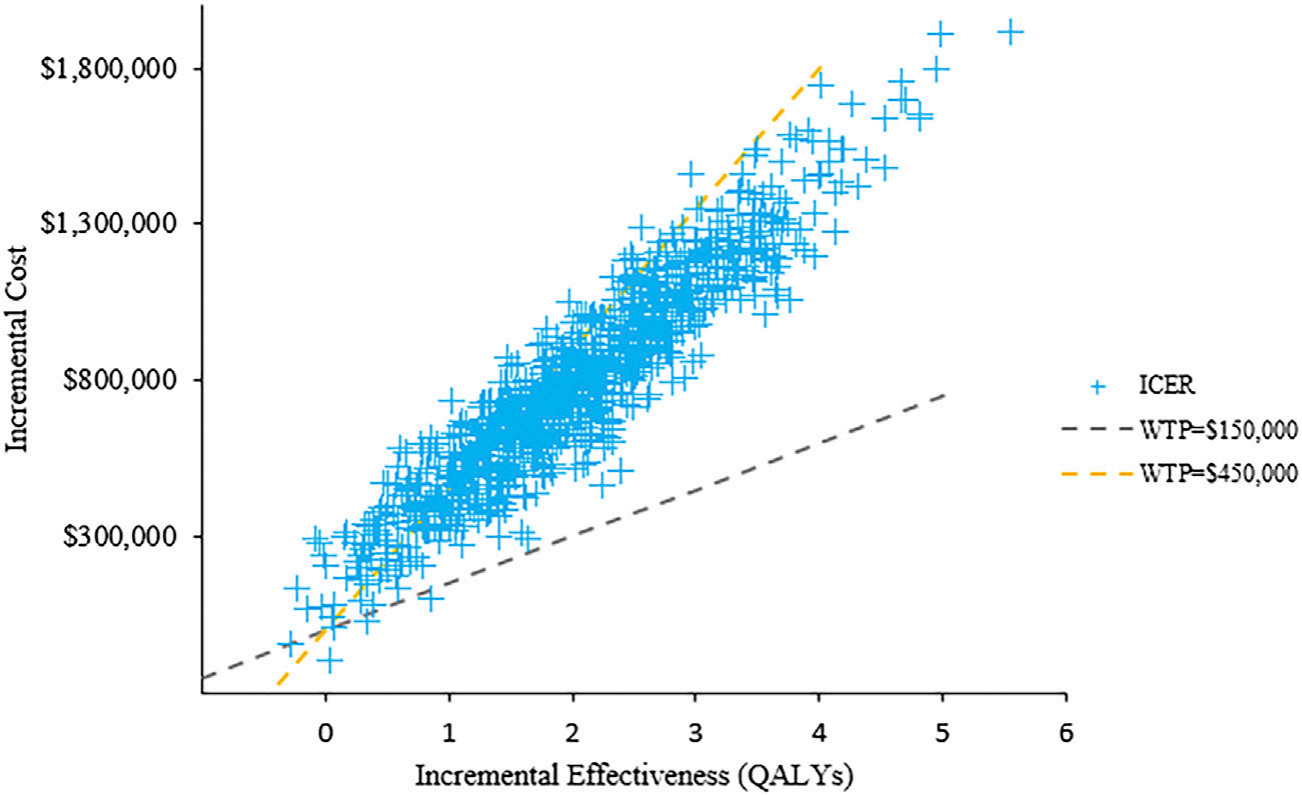
Scatter plot of probabilistic sensitivity analysis. ICER: incremental cost-effectiveness ratio; WTP: willingness to pay.

The results of PSA with 1,000 replicated Monte Carlo simulations were showed in Figures 3, 4. It suggested that the acceptability of D-VMP was 0% when the WTP for per QALY was $150,000. In order to explore the cost-effectiveness of D-VMP, we increased WTP to $450,000/QALY, and the acceptability of D-VMP and VMP were 0.83 and 0.17 respectively (Figures 3, 4).

## Discussion

Although the D-VMP regimen had better efficacy than VMP in TNE NDMM, it was costly. We conducted a long-term economic evaluation of adding daratumumab to VMP as first-line therapy in TNE NDMM. The base case analysis results showed that the LYs and QALYs gained of D-VMP vs. VMP were 3.00 and 2.03, respectively. The results of cost-effectiveness were estimated at $262,526 per LY and $388,364 per QALY. Sensitivity analysis showed within the range of parameters changed, the ICERs remained > $150,000. All above indicated that D-VMP regimen was not cost-effectiveness.

This study was the first cost-effectiveness analysis of daratumumab-based regimens for TNE NDMM. Therefore, it cannot be compared with other results of similar studies. There were only a few cost-effectiveness studies on daratumumab-based treatments of RRMM. Zeng et al. (Zeng et al., 2020) performed an economic evaluation of adding daratumumab to bortezomib and dexamethasone in RRMM, the result showed that the ICER for daratumumab plus bortezomib and dexamethasone (DVd) compared to bortezomib and dexamethasone (Vd) was $213,164/QALY in base case analysis. In their subgroup analysis, DVd regimen with WTP of $200,000/QALY was more cost-effective than Vd, the condition was patients who were first time receiving second-line treatment, treatment-free interval >6 months or >12 months, in International Staging System (ISS) Stage I disease, standard cytogenetic risk, or had received thalidomide. Their sensitivity analysis results showed that when the price of daratumumab was reduced to less than $47 (10 mg), it was cost-effective when the WTP threshold was $200,000. If the WTP threshold was $150,000, the price of daratumumab need to be reduced to less than $25 (10 mg). Zhang et al. (Zhang et al., 2018) conducted a cost-effectiveness of daratumumab-based triple therapy in RRMM from the perspective of US payers and found that the ICER of daratumumab plus lenalidomide and dexamethasone (DVd) was $284,180 per QALY relative to Vd; the ICER of DRd was $1,369,062 per QALY relative to Rd. They concluded that when daratumumab is reduced to 37% of the current price, DVd would be cost-effective if the WTP was $50,000/QALY. In the economic evaluation of treatments in R/RMM, Carlson et al. (Carlson et al., 2018) conducted a cost-effectiveness analysis based on network meta-analysis results from the perspective of US payers, and found that the expected LYs daratumumab-based regimens (daratumumab range: 6.71–7.38; non-daratumumab range: 3.25–5.27) and QALYs (daratumumab range: 4.38–5.44 vs non-daratumumab range: 2.04–3.46) were higher than non-daratumumab-based regimens. They concluded that the daratumumab-based regimens for second- and third-line RRMM may provide clinical benefits by prolonging PFS and OS and improving quality of life, but the price of daratumumab need to be reduced to benefit more patients. In the heavily pretreated RRMM patients, Pelligra et al. and Gong CL et al. (Pelligra et al., 2017; Gong et al., 2019) came to different conclusions. Gong CL et al. reported that the ICER of daratumumab gained vs pomalidomide was $156,385 per QALY, but Pelligra et al. reported that the QALYs of daratumumab gained was lower than pomalidomide but the costs higher than its. Despite the limited number of studies, most studies showed that daratumumab-based regimens had higher costs but limited clinical benefits. These results suggested that daratumumab need reduce price to be cost-effective.

There are some limitations in our study. First, ALCYONE (Mateos et al., 2018; Mateos et al., 2020) is a large-scale multi-center randomized controlled trial, but it is also the only trial that revealed the efficacy and safety of adding daratumumab to VMP. Our model was closely related toresults of ALCYONE, so the biases within this trial may affect our results. Second, the current follow-up results of ALCYONE had not been extended the entire life year of all patients, therefore, the limitation of using Weibull distribution to extrapolate the survival curve of patient’s lifetime is inevitable. Third, we made assumptions about the data that were lacking. Although sensitivity analysis was conducted within a certain range, it may still affect our results. Fourth, utilities of our study were estimated based on the previously published literature, not the real utilities of patients in ALCYONE due to the lack of utility data. Fifth, we assumed that the patients only received first-line and second-line treatments, but the actual situation of patient’s medication is very complicated.

## Conclusion

From the perspective of US payers and based on $150,000 as a willingness to pay threshold, adding daratumumab to bortezomib, melphalan and prednisone for untreated multiple myeloma was estimated not to be cost-effective.

## Data Availability

The original contributions presented in the study are included in the article/Supplementary Material, further inquiries can be directed to the corresponding authors.
